# Overview of waterfowl circovirus: research progress and future perspectives

**DOI:** 10.3389/fvets.2026.1871872

**Published:** 2026-06-15

**Authors:** Mengyan Zhang, Zhongjun Tian, Shoukang Ma, Yu Huang, Qizhang Liang, Guanghua Fu, Chunhe Wan, Guangwen Yin

**Affiliations:** 1 College of Animal Sciences, Fujian Agriculture and Forestry University, Fuzhou, China; 2 Fujian Key Laboratory for Avian Disease Control and Prevention, Institute of Animal Husbandry and Veterinary Medicine, Fujian Academy of Agricultural Sciences, Fuzhou, China

**Keywords:** diagnostic technology, duck circovirus (DuCV), genomic characteristics, goose circovirus (GoCV), vaccines, waterfowl circovirus

## Abstract

Waterfowl circovirus (WFCV) is an important member of the genus Circovirus within the family Circoviridae, encompassing duck circovirus (DuCV) and goose circovirus (GoCV). As a pathogen that poses a serious threat to the global waterfowl industry, WFCV induces significant immunosuppression in its hosts, leading to growth retardation, feather abnormalities, and increased susceptibility to other pathogens. Consequently, it results in markedly elevated mortality rates among affected birds and causes substantial economic losses. In recent years, with the advancement of molecular biology techniques, notable progress has been made in research on waterfowl circoviruses. This review aims to systematically consolidate current findings on the genomic characteristics, pathogenic mechanisms, and clinical manifestations of WFCV while also summarizing the existing detection technologies and the status of vaccine development. Future research directions and prevention strategies are proposed, with the goal of providing a scientific reference for the effective control of this disease.

## 1 Introduction

The waterfowl industry plays a significant role in the global agricultural economy; however, the outbreak and spread of various infectious diseases persistently threaten its healthy development. Among these pathogens, waterfowl circovirus (WFCV), an emerging DNA virus, has become a serious threat to the waterfowl industry worldwide. According to the latest classification by the International Committee on Taxonomy of Viruses (ICTV), the family *Circoviridae* is divided into two genera: *Circovirus* and *Cyclovirus*. Both duck circovirus (1) and goose circovirus (2) are members of the genus *Circovirus*, while WFCV serves as a collective term for these two viruses. Based on the sequence differences in the ORF3 and Cap proteins, DuCV can be classified into two genotypes, DuCV-1 and DuCV-2, which are further divided into seven subtypes: DuCV-1a, DuCV-1b, DuCV-1c, DuCV-1d, DuCV-2a, DuCV-2b, and DuCV-2c (3, 4). Currently, DuCV-1 is more widely distributed than DuCV-2, and studies have identified a common evolutionary origin for the two genotypes (3, 5). In 2022, a novel DuCV, tentatively classified as DuCV-3, was identified in laying ducks in Hunan Province, China. DuCV-3 does not cluster with the previously known DuCV-1 and DuCV-2 but exhibits greater similarity to GoCV (6). The complete genome of GoCV was first cloned and sequenced in 2001 (2), and subsequent reports on GoCV have emerged worldwide (7–12). Based on phylogenetic analysis, GoCVs can be broadly divided into three distinct genetic groups, among which Groups I and II can each be further subdivided into two subgroups (I1, I2, and II1, II2, respectively) (13).

WFCV infection can trigger a range of clinical symptoms. Affected animals typically exhibit growth retardation, diarrhea, and poor feather quality or feather loss. However, its most defining characteristic lies in its ability to induce significant immunosuppression in the host (14, 15), which severely impairs the resistance of infected animals to other pathogens and increases their susceptibility to secondary infections (16, 17). Recently, several cases of cross-species transmission involving WFCV have also been reported (18, 19), further complicating research on viral transmission and increasing the difficulty of disease prevention and control.

Current research on WFCV remains in a relatively primary stage. In particular, there remain numerous questions awaiting resolution regarding the mechanisms of virus–host interactions and the development of effective prevention and control strategies. This review aims to systematically consolidate and analyze the research progress on waterfowl circoviruses, provide an in-depth analysis of their genomic characteristics and epidemiological status, summarize the current state of diagnostic techniques and vaccine development, and propose future research directions and prevention strategies, with the goal of providing a theoretical basis for the scientific control of this disease.

## 2 Genomic characteristics of WFCV

### 2.1 Overview of viruses

**Figure 1:**
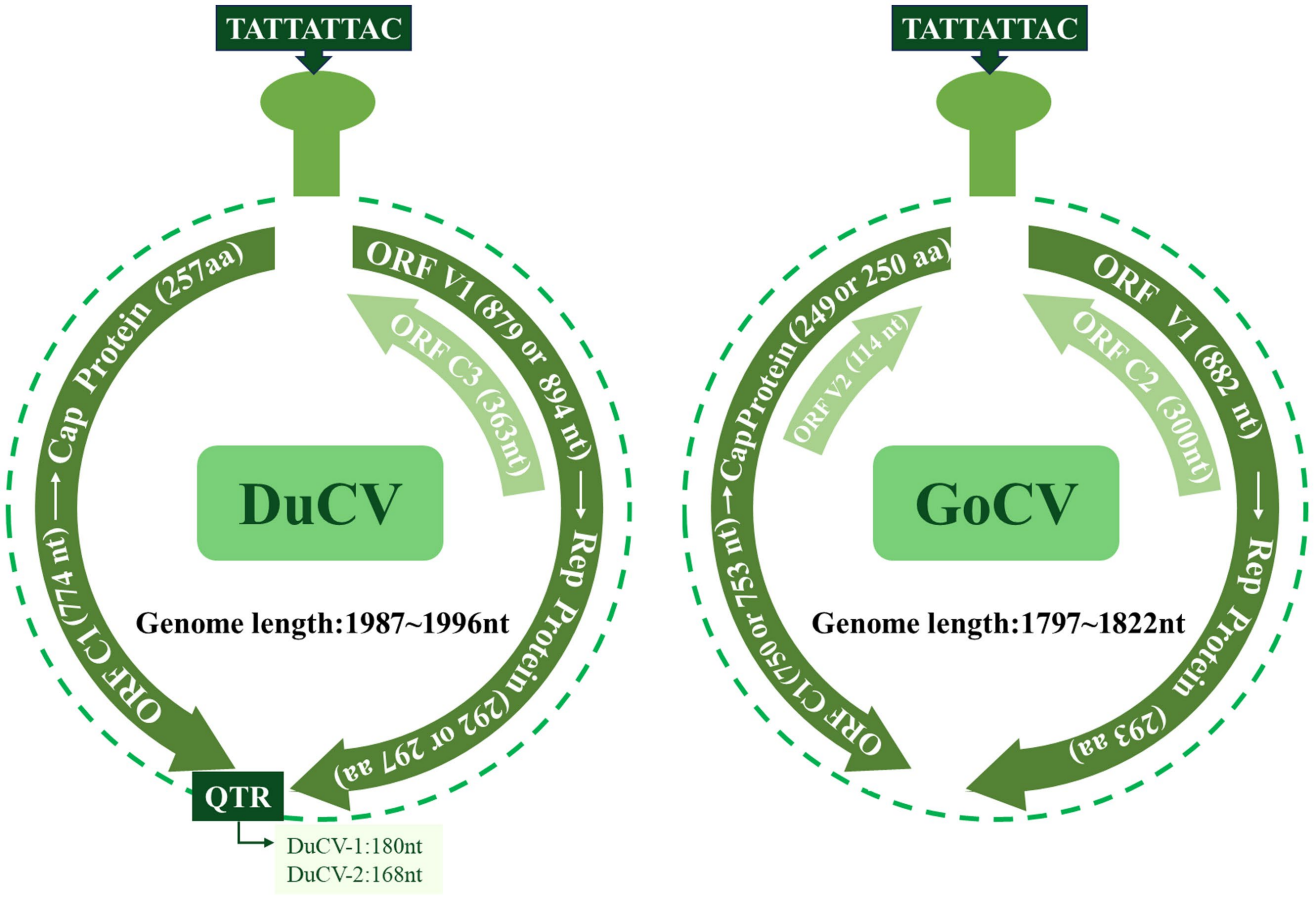
Genome organization of DuCV and GoCV.

The genome of waterfowl circovirus is a typical covalently closed, single-stranded circular DNA (Figure 1), a hallmark feature of viruses in the family Circoviridae (20). This circular structure endows the genome with high stability. Although the genome sizes of different waterfowl circovirus strains vary to some extent, they generally remain within a very small range (11, 21). A defining characteristic of the circovirus genome is its ambisense organization, meaning that the viral coding genes are distributed on both strands of the DNA circle and are transcribed in opposite directions. This indicates that circoviruses require proteins encoded by both the viral sense strand and the complementary strand for replication (22). This strategy enables the virus to encode multiple proteins within a very small genome, maximizing the utilization of genetic information within the viral genome.

### 2.2 Open reading frame (ORF) and its functions

The majority of known circovirus genomes, including that of WFCV, contain two major, well-characterized open reading frames, ORF1 and ORF2 (1, 23). ORF1 encodes the viral replication-associated protein (Rep). The Rep protein is the sole enzyme encoded by WFCV that participates in viral DNA replication, representing the most important nonstructural protein in the viral life cycle and exhibiting a high degree of conservation (7). Rep is a multifunctional protein; its N-terminal region typically contains conserved domains involved in initiating rolling circle replication, which are responsible for recognizing and cleaving the replication origin. Its C-terminal region contains a helicase-like domain, possessing ATPase and DNA helicase activities that enable it to unwind double-stranded DNA during replication. Consequently, the Rep protein integrates the functions of recognition, binding, cleavage, and unwinding, serving as the core component in initiating and driving viral rolling circle replication (24, 25). ORF2 encodes the viral capsid protein (Cap), which is the sole structural protein of the virus. The Cap protein is responsible for assembling into the icosahedral capsid that encloses the viral genome, forming infectious viral particles, and exhibits a relatively high mutation rate (7). In addition to its structural function, the Cap protein plays multiple roles in viral pathogenesis. It serves as the major antigen on the surface of circoviruses and is capable of inducing neutralizing antibodies in the host, making it a primary target for vaccine design. Simultaneously, the Cap protein is directly involved in virus–host cell interactions, such as mediating viral attachment to and entry into host cells. Studies have shown that the Cap protein itself is a key pathogenic determinant of the virus, and its interaction with the host immune system is directly linked to the occurrence of immunosuppression.

With the advancement of sequencing technologies and bioinformatics analysis tools, researchers have identified other potential smaller open reading frames (ORFs) beyond ORF1 and ORF2 in the genomes of WFCV and other circoviruses. Although the products of these ORFs may not be essential for viral replication in *in vitro* cell cultures, they are likely to play important regulatory roles during *in vivo* infection, particularly in viral pathogenesis and immune evasion. In the genome of DuCV, the presence of ORF3 has been identified. Studies have shown that the ORF3 of DuCV encodes a protein capable of inducing apoptosis (26), which may primarily activate the apoptotic pathway in duck embryo fibroblasts (DEFs) through the mitochondrial pathway (27). This function is highly analogous to the well-characterized ORF3 protein of porcine circovirus type 2 (PCV2). The ORF3 protein of PCV2 has been confirmed as a key pro-apoptotic factor; it induces apoptosis in infected cells and lymphocytes by activating critical apoptotic molecules such as caspase-8 and caspase-3, thereby leading to lymphocyte depletion in immune organs, which constitutes one of the core mechanisms of PCV2 pathogenesis. Based on existing functional studies of the PCV2 ORF3 protein, it is speculated that the DuCV ORF3 protein, as an important virulence-associated factor, may induce apoptosis in immune cells following infection through a similar mechanism, thereby contributing to the development of immunosuppression. However, this speculation has not yet been supported by relevant reports, and further in-depth analysis and validation are required.

In addition to ORF3, more in-depth genomic analyses have predicted the presence of additional potential ORFs. For instance, some studies have identified up to six major ORFs (designated ORF-V1 to V3 and ORF-C1 to C3) or four major ORFs in certain circovirus genomes. In current research on porcine circovirus (PCV), ORF4 and ORF5 have also been identified. The functions of these small ORFs remain largely unclear and are the subject of considerable debate. These findings suggest that similar ORFs may also exist in the WFCV genome. In-depth investigation into the functions of these ORFs will contribute to a more comprehensive understanding of how the virus finely regulates host cellular processes to achieve replication and spread, thereby providing new theoretical support for elucidating the complete pathogenic mechanisms of the virus.

### 2.3 Genomic elements

The initiation of viral genome replication is a critical step in the viral life cycle. Within the WFCV genome, there exists a highly conserved stem–loop structure, which is typically located in a noncoding region, namely, the intergenic region (IGR) (28). This stem–loop structure contains a conserved nonanucleotide sequence that serves as the origin of replication for rolling circle replication (RCR) of the viral genome. The replication origin of WFCV is characterized by its high conservation and structural specificity, and its integrity is essential for successful viral replication. Furthermore, this conserved sequence is commonly used as a universal marker for determining the starting site and orientation of circovirus genomic sequences.

Other studies have shown that a quadruple tandem repeat (QTR) sequence exists between the Rep and Cap genes in the DuCV genome, which can enhance mRNA stability (29). Deletion of this QTR significantly reduces viral transcription and replication. The uniqueness and evolutionary conservation of the QTR across genotypes 1 and 2 make it a molecular marker for DuCV genotyping (30).

## 3 Pathogenic mechanism and clinical symptoms

### 3.1 Pathogenic mechanism

The most significant clinical and pathological consequence of waterfowl circovirus infection is severe immunosuppression. This comprehensive impairment of immune function is the primary cause of mortality and economic losses. The underlying molecular mechanisms involve viral interference with and disruption of multiple aspects of the host’s innate and adaptive immune responses. Due to the limited direct research on WFCV, the current understanding of its potential immunosuppressive mechanisms is primarily derived from studies on related members within the same genus (15).

#### 3.1.1 Interference with innate immunity

Innate immunity serves as the first line of defense against viral infection, with the type I interferon (IFN-I) system playing a central role. Circoviruses have evolved various strategies to antagonize the production and signaling of IFN-I. Studies have shown that multiple proteins of PCV can act on various key nodes within the signaling pathway to inhibit IFN-I production at the source (31, 32). In addition, blocking IFN-I signaling is another employed strategy (33); in this manner, even if only a small amount of IFN-I is produced, the virus can still block its downstream antiviral effects. As a virus belonging to the same genus as PCV, whether WFCV employs similar strategies to inhibit the production and signaling of type I interferons (IFN-I) warrants further investigation.

Another strategy involves modulating the cytokine network. In addition to affecting the interferon system, WFCV infection can also lead to dysregulation of the cytokine network. Studies have demonstrated that following DuCV infection, the binding of the Cap protein to CLDN2 triggers the intracellular MAPK–ERK pathway and activates the downstream transcription factor SP5, thereby upregulating the expression of both SP5 and CLDN2. Mature CLDN2 is then transported to the cell surface, increasing viral adherence to target organs and promoting viral infection and transmission (34). This suggests that viral proteins may interact with host cellular receptors to activate or inhibit signaling pathways such as PI3K/Akt, NF-κB, and MAPK, thereby finely regulating the expression of cytokines.

#### 3.1.2 Destruction of adaptive immunity

Adaptive immunity is crucial for clearing viral infections and establishing long-term immunological memory. WFCV primarily dismantles the host’s adaptive immune system by directly attacking and destroying immune cells. Similar to PCV, WFCV exhibits marked lymphotropism, allowing it to target and infect key immune cells, including lymphocytes (T cells and B cells), monocytes, and macrophages (35, 36). Direct infection and destruction of these cells represent the fundamental cause of immunosuppression. Furthermore, numerous studies have confirmed that circovirus infection induces extensive lymphocyte apoptosis, which is the primary driver of atrophy in immune organs such as the thymus, bursa of Fabricius, and spleen, as well as lymphocyte depletion (36, 37). Additionally, other viral proteins or cellular stress induced by the infection itself may activate apoptotic pathways through various mechanisms. This indiscriminate destruction of lymphocytes severely compromises both cellular and humoral immunity.

### 3.2 Clinical symptoms

The most significant clinical consequence of WFCV infection is immunosuppression (38). Clinically, waterfowl infected with WFCV often present with depression, emaciation, ruffled feathers, and poor growth, which directly reflect virus-induced immunosuppression and chronic wasting processes (37). The virus primarily targets the host’s immune organs (39); the thymus, bursa of Fabricius, and spleen of infected animals exhibit pronounced atrophy visible to the naked eye. In these organs, lymphocytes undergo diffuse or focal necrosis and depletion, the structure of lymphoid follicles is disrupted, the boundary between the cortex and medulla becomes indistinct, and macrophage infiltration along with inclusion body formation can be observed. These irreversible lesions serve as morphological evidence of impaired immune function. This direct destruction of the immune system significantly compromises the infected animal’s resistance to other pathogens, such as bacteria, fungi, or other viruses, leading to various secondary and mixed infections, which are the primary reasons for the clinical complexity and increased mortality.

## 4 Epidemiological investigation

### 4.1 Discovering history and geographic distribution

The discovery of waterfowl circoviruses is relatively recent. Goose circovirus (GoCV) was first identified in goose flocks in Germany in 1999 (40), while duck circovirus (DuCV) was subsequently reported in duck flocks in Germany in 2003 (1). Since then, both viruses have been successively detected in waterfowl farming regions across continents, indicating that they have become a global epidemic.

As the region where WFCV was first reported, Europe continues to experience widespread circulation of the virus. A study from Poland indicated that GoCV is prevalent in commercial goose flocks and is often associated with coinfections with other pathogens, thereby exacerbating the severity of clinical disease (10). Asia, as the global center of waterfowl farming, is also the region where research on WFCV is most concentrated. As the world’s largest producer of ducks and geese, China faces a particularly severe WFCV epidemic. Surveillance data from recent years show a very high prevalence of WFCV in China (41). One study reported that the seroprevalence of GoCV in goose flocks from Guangdong, Shandong, and Fujian provinces reached as high as 71.06% (42). In duck populations, the prevalence of DuCV is similarly widespread; a 2022 study found an overall prevalence of 35.5% for GoCV in domestic geese in Guangdong Province (11). The presence of DuCV has also been confirmed in countries such as Vietnam (20), South Korea (43), and Thailand (44), with genetic characterization studies indicating that WFCV may be prevalent throughout Asia. Although North America is also a major poultry-producing region, publicly available data on the recent prevalence and distribution of WFCV in this area remain relatively limited.

### 4.2 Host animals and transmission dynamics

The primary hosts of WFCV are domestic ducks and geese. However, a notable feature of its epidemiology is the occurrence of cross-species transmission and coinfection. Studies have found that not only GoCV but also DuCV can be detected in goose flocks, and vice versa (18, 45). In some cases, both GoCV and DuCV can even be detected simultaneously within the same individual. This diversity in viral ecology poses significant challenges for disease diagnosis and control and suggests that frequent genetic exchange and recombination among these viruses may be occurring, thereby accelerating their evolution (46).

The primary route of WFCV transmission is considered to be horizontal, occurring through the digestive tract via contact with virus-contaminated feces, feed, water, and the environment. Viral particles exhibit high environmental stability, facilitating their spread within and between farms. The possibility of vertical transmission, i.e., transmission from parent to offspring through hatching eggs, has also been suggested. Currently, evidence supporting the transmission routes of DuCV has been documented (36, 47, 48). However, the transmission mechanisms of GoCV warrant further investigation.

### 4.3 Main risk factors

The epidemiology of WFCV is influenced by a combination of factors. First, many small-scale farms operate with relatively extensive management practices and inadequate biosecurity measures, which facilitate virus transmission. Conversely, high-density intensive farming models, once the virus is introduced, can also lead to rapid and widespread dissemination. Furthermore, the immunosuppression induced by WFCV predisposes infected animals to other pathogens (49). Clinically, WFCV is frequently associated with coinfections involving parvoviruses (19), adenoviruses (50), *Escherichia coli* (51), and *Salmonella* (52), resulting in complicated clinical presentations and increased mortality. Notably, young waterfowl, with their underdeveloped immune systems, exhibit more severe clinical symptoms following infection (49). Regarding long-distance transmission, the trade of live poultry and the global movement of poultry products likely play critical roles (11). Additionally, wild waterfowl may serve as potential viral reservoirs (53). Their migratory activities could also contribute to the cross-regional spread of WFCV, although research in this area remains limited.

## 5 Diagnostic technology

### 5.1 Serological testing methods

Serological methods detect virus-specific antibodies in serum to determine whether an animal has been previously infected, serving as an essential tool for large-scale epidemiological investigations, immune status assessment, and vaccine efficacy evaluation. Enzyme-linked immunosorbent assay (ELISA) is the most widely used technique among enzyme immunoassays. Commonly employed ELISA formats include the double-antibody sandwich method, used for detecting large-molecule antigens, and the indirect method, used for detecting specific antibodies. The indirect ELISA developed based on recombinant Cap protein represents the predominant serological detection method (42, 54, 55). Researchers have established highly specific and reproducible ELISA methods by preparing high-purity recombinant Cap protein as a coating antigen using prokaryotic or eukaryotic expression systems. In addition, an indirect immunofluorescence assay has been developed for detecting virus-specific serum antibodies (56). These methods are suitable for large-scale screening, providing robust technical support for assessing the epidemiological breadth of WFCV within a region.

### 5.2 Molecular biology detection methods

Molecular diagnostic techniques detect infection by identifying the nucleic acids of pathogens, offering extremely high sensitivity and specificity, and are currently considered the gold standard for etiological diagnosis. A variety of diagnostic methods based on polymerase chain reaction (PCR) have been developed (57, 58). These techniques are relatively simple to perform and are suitable for most laboratories equipped with basic molecular biology facilities. For instance, real-time quantitative PCR (real-time qPCR) enables real-time monitoring of the amplification process and precise quantification of products by incorporating fluorescent dyes (e.g., SYBR Green I) (59) or specific probes (e.g., TaqMan) (60, 61) into the reaction system. This technique not only offers exceptional sensitivity but also allows for quantitative analysis of viral load, facilitating the assessment of infection severity. Furthermore, to address the complexity of cross-species and coinfections, researchers have developed multiplex PCR techniques (62–66). These methods enable the simultaneous detection of multiple pathogens, thereby improving detection efficiency and reducing costs, making them well suited for large-scale epidemiological screening.

However, PCR technology relies on precise temperature control equipment, which presents certain limitations. Isothermal amplification techniques, which can complete nucleic acid amplification at a constant temperature, are characterized by rapidity and simplicity, making them an ideal platform for developing next-generation point-of-care rapid detection reagents. Loop-mediated isothermal amplification (LAMP) utilizes 4–6 specific primers for efficient amplification under isothermal conditions, with results determined through turbidity, fluorescence, or color change (67). Recombinase-aided amplification (RAA) technology mimics the *in vivo* DNA recombination and repair process, using recombinase, single-stranded binding protein, and DNA polymerase to achieve rapid nucleic acid amplification at 37–42 °C *in vitro*. One study developed a recombinase-aided amplification-lateral flow dipstick (RAA-LFD) detection method, enabling visual interpretation of results by incorporating lateral flow dipstick (LFD) technology (68, 69). Additionally, a real-time fluorescence recombinase-aided amplification (RF-RAA) method for DuCV detection has been successfully developed (70), demonstrating good specificity, requiring no specialized equipment, and offering ease of operation, making it suitable for on-site rapid screening in farms.

## 6 Vaccine research and development status

### 6.1 Traditional vaccine technology

Inactivated vaccines and live attenuated vaccines represent classic approaches in traditional vaccine development. Studies have explored the development of inactivated vaccines against DuCV, with preliminary efficacy evaluations conducted in Muscovy ducks (71). However, the production of inactivated vaccines relies on the large-scale *in vitro* cultivation of the virus, and the poor adaptability of WFCV to cell lines makes it difficult to achieve high-titer viral propagation, which significantly limits the production capacity of inactivated vaccines and drives up manufacturing costs. Although a stable primary goose embryo kidney (GEK) cell culture system for GoCV propagation has been successfully established (72), enabling sustained viral replication and the development of a standardized gosling infection model, the feasibility and safety of applying this method to vaccine production still require further investigation.

### 6.2 New vaccine technology

With the rapid advancement of vaccine technology, a variety of novel vaccine platforms have opened new possibilities for the development of WFCV vaccines. Viral vector vaccines utilize harmless or attenuated viruses as vectors to deliver antigenic genes of the target pathogen into the host. For waterfowl, well-established avian viral vectors, such as fowlpox virus, Newcastle disease virus, or duck enteritis virus, could be employed to construct recombinant viral vector vaccines expressing the WFCV Cap protein. This strategy not only effectively induces immunity but also holds the potential for developing multivalent combination vaccines, enabling protection against multiple diseases with a single vaccination—an approach of great significance for simplifying immunization protocols on farms.

Subunit vaccines represent the most promising direction for development. The core principle of this approach involves utilizing genetic engineering techniques to mass-produce the key immunogenic protein of the virus, the capsid protein, in prokaryotic or eukaryotic expression systems, which is then purified and used as an antigen to formulate the vaccine. The Cap protein is the primary target for inducing protective neutralizing antibodies and can self-assemble into virus-like particles (VLPs). VLPs closely resemble native viruses in morphology and antigenicity but lack genetic material, rendering them both safe and highly immunogenic. Current studies have successfully expressed recombinant Cap protein for DuCV, providing a foundation for the preparation of subunit vaccines (73).

Nucleic acid vaccines are divided into DNA vaccines and mRNA vaccines. Their advantages include extremely rapid design and production, as well as the ability to induce robust T-cell and B-cell immune responses. DNA vaccines involve constructing a gene encoding a specific protein into a plasmid vector, which is then directly inoculated into the animal. Host cells express antigen proteins, thereby eliciting both humoral and cellular immunity. Currently, three DNA vaccines encoding the DuCV Cap protein have been developed based on the eukaryotic vector pcDNA3.1; all three were able to induce significant specific immune responses, with the DNA vaccine encoding the Cap protein lacking the NLS but containing the tPA signal sequence demonstrating superior immunogenicity in experiments (74). In contrast, mRNA vaccines operate on a similar principle to DNA vaccines in terms of inducing immunity, but they deliver messenger RNA encoding the antigen instead. They offer advantages such as rapid development and a high safety profile.

## 7 Future prospects

As a globally distributed pathogen capable of inducing severe immunosuppression, waterfowl circovirus has posed a serious constraint on the healthy development of the waterfowl industry. Future research should prioritize elucidating the molecular mechanisms underlying viral replication and pathogenesis while also exploring the impact of coinfection and environmental factors on disease manifestation. In the face of the formidable challenges posed by waterfowl circovirus disease, establishing rapid and accurate diagnostic methods and developing effective prevention and control strategies are also critical. The diagnosis of WFCV primarily relies on molecular biology and serological techniques; rapid, sensitive, and specific detection methods are essential for early disease detection, epidemiological surveillance, and eradication efforts. Although a variety of diagnostic techniques have been developed, information on point-of-care rapid diagnostic kits for waterfowl circovirus that have received regulatory approval and achieved large-scale commercialization remains very limited. Portable rapid diagnostic products represent the future direction for development.

Vaccination is considered the most economical and effective strategy for preventing and controlling WFCV infection. However, current vaccine development for WFCV remains in the early stages of exploration. The successful development of a WFCV vaccine still faces several major obstacles: (1) The difficulty of isolating and cultivating WFCV in conventional cell lines represents a fundamental bottleneck limiting the development of inactivated and live attenuated vaccines. (2) Although the mutation rate of WFCV as a DNA virus is lower than that of RNA viruses, it still undergoes continuous evolution, which may lead to immune evasion, necessitating ongoing surveillance and updating of vaccine strains. (3) The immunosuppressive nature of WFCV imposes higher demands on vaccine efficacy; the vaccine must establish robust protection before the virus causes irreversible damage. (4) Currently, there is a lack of standardized efficacy evaluation criteria and animal models for WFCV vaccines, leaving the assessment of vaccine effectiveness in need of further refinement.

With the advancement of molecular biology, immunology, genomics, and other technologies, interdisciplinary collaboration will expand our understanding of waterfowl circoviruses and facilitate the development of simpler, more rapid diagnostic techniques as well as safer, more effective next-generation vaccines. Ultimately, this will enable effective control of waterfowl circovirus disease, providing a solid technological foundation for the healthy and sustainable development of the global waterfowl industry.
